# Optimization of Regeneration and *Agrobacterium*-Mediated Transformation Protocols for Bi and Multilocular Varieties of *Brassica rapa*

**DOI:** 10.3390/plants12010161

**Published:** 2022-12-29

**Authors:** Uzair Muhammad Khan, Nabeel Shaheen, Ayesha Farooq, Rizwana Maqbool, Sultan Habibullah Khan, Muhammad Tehseen Azhar, Iqrar Ahmad Rana, Hyojin Seo

**Affiliations:** 1 Center for Advanced Studies in Agriculture and Food Security, University of Agriculture, Faisalabad 38040, Pakistan; 2 Department of Plant Breeding and Genetics, University of Agriculture, Faisalabad 38040, Pakistan; 3 Centre of Agricultural Biochemistry and Biotechnology, University of Agriculture, Faisalabad 38040, Pakistan; 4 Korea Soybean Research Institute, Jinju 52840, Republic of Korea

**Keywords:** genetic transformation, induction media, flowers, *B. rapa*, *Agrobacterium*, in-planta, tissue culture

## Abstract

The regeneration of the high-yielding multilocular types has not been attempted, although successful regeneration and transformation in brassica have been done. Here, we report efficient regeneration and transformation protocols for two *B. rapa* genotypes; UAF11 and Toria. The *B. rapa* cv UAF11 is a multilocular, non-shattering, and high-yielding genotype, while Toria is the bilocular type. For UAF11 8 shoots and for Toria 7 shoots, explants were observed on MS supplemented with 3 mg/L BAP + 0.4 mg/L NAA + 0.01 mg/L GA_3_ + 5 mg/L AgNO_3_ + 0.75 mg/L Potassium Iodide (KI), MS salt supplemented with 1 mg/L IBA and 0.37 mg/L KI produced an equal number of roots (3) in UAF11 and Toria. For the establishment of transformation protocols, *Agrobacterium*-mediated floral dip transformation was attempted using different induction media, infection time, and flower stages. The induction medium III yielded a maximum of 7.2% transformants on half-opened flowers and 5.2% transformants on fully opened flowers in UAF11 and Toria, respectively, with 15 min of inoculation. This study would provide the basis for the improvement of tissue culture and transformation protocols in multilocular and bilocular Brassica genotypes.

## 1. Introduction

Oilseed *Brassicas* are classified into two classes, Rapeseeds (*B. napus*) and Mustards (*B. juncea*, *B. rapa*, and *B. carinata*), and rank at third position after soybean (*Glycine max*) and palm (*Elaeis guineensis*) in global vegetable oil production [1,2]. It is projected that *Brassica* yield could increase to 101.43 MMT (Million Metric tons) in 2022–2023 which would be 10% more than the previous years’ production. However, such an increase is limited by the genetic potential, which is still not well explored in this group. Reduction of 58.3% and 31% yield losses due to heat and drought stress highlights the need for the development of climate-resilient *Brassicas*. Moreover, the adverse effects of these factors are not limited to seed yield loss but could also lower the seed oil contents as well [3].

*Brassicas* are known for their genetic diversity. The genotypes with the superior phenotype, like multilocular types (Multiple locules in silique), could be a good addition to high-yielding genotypes. In the past, direct association of yield with multilocular-type siliques was identified [4,5,6]. Multilocular types could produce 30% more yield compared to bilocular (Two locules per silique) genotypes [4]. Along with desirable phenotypes, multilocular types carry some undesirable characteristics that need to be improved. These traits include high erucic acid and glucosinolates and low adaptability to diversified climates [7]. Conventional breeding is widely adopted to improve crop plants. However, it is time-consuming, labor-intensive, and needs large populations to apply any breeding strategy. Modern biotechnological approaches (transgenics development and genome editing), on the other hand, are rapid and should be adopted to combat these issues efficiently [8]. Establishing a transformation protocol is a crucial and elementary step before attempting genome editing or transgenic development. There are already some regeneration and transformation protocols reported in different *Brassica* species, but these are restricted to specific genotypes only due to genotypic specificity [9,10,11,12,13]. Furthermore, the success of plant tissue culture is dependent on several factors, which include genotype, age of explant, type of explant, and combinations of growth regulators [14,15]. The regeneration of *Brassicas* almost always remains under question due to their different genetic backgrounds [16,17].

The *Agrobacterium*-mediated floral dip transformation protocol was first reported by Clough and Bent [18] in *Arabidopsis thaliana*. In the past, *A. thaliana* floral dip transformation efficiency was reported from 0.04 to 14% [19,20]. Nowadays, *Agrobacterium*-mediated transformation protocols have gained much more importance due to their success and efficiency in plants. This is due to their less labor intensiveness and cost-effectiveness [21,22]. In previous studies, the non-responsiveness of *B. rapa* to the regeneration protocol was also reported [10,23]. To overcome this barrier, a parallel in-planta transformation approach was evaluated.

In this study, the regeneration was attempted for multilocular and bilocular genotypes under the *Agrobacterium*-mediated transformation stress. The varying BAP and IBA concentrations were evaluated for optimization of shooting and rooting response. In parallel floral dip, *Agrobacterium*-mediated transformation was also performed to overcome the regeneration barrier. This experiment involved the evaluation of different induction media, flower stages, and time durations to optimize the variables for transforming multilocular genotypes. This study would help plant scientists to understand the response of *B. rapa* multilocular type genotypes against regeneration and *Agrobacterium* stress. Optimized genetic transformation protocols could be used in the future to improve/change any desired trait through genome editing, e.g., gene silencing and overexpression.

## 2. Results

### 2.1. In-Vitro Regeneration

**Figure 1:**
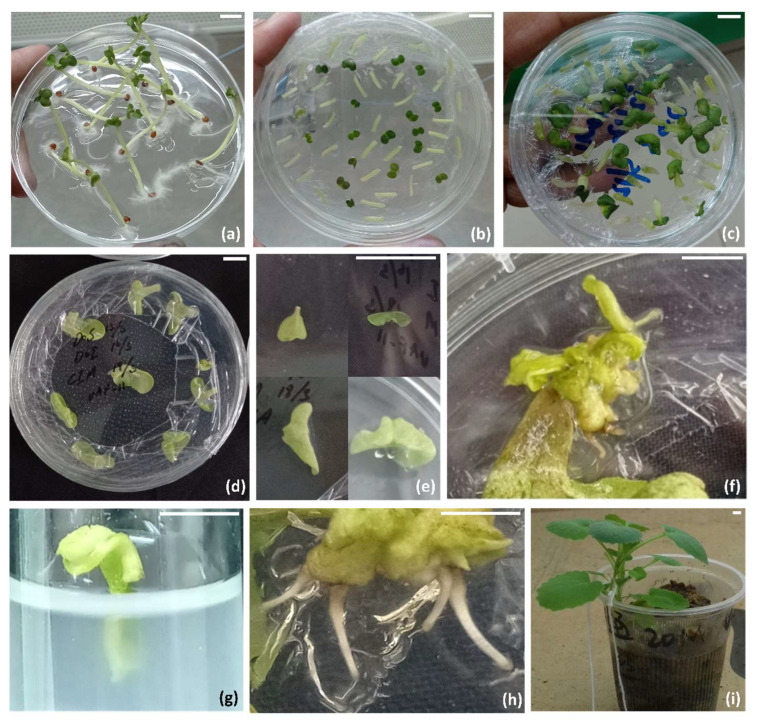
Regeneration and acclimatization of UAF11 and Toria. (**a**) In-vitro grown seedlings of UAF11 and Toria, (**b**) excised cotyledonary leaves and hypocotyls on cocultivation medium, (**c**) explants on the callus induction medium, (**d**) cotyledons on regeneration media, (**e**) progressive increase in the swelling of the cotyledons after *Agrobacterium* cocultivation, (**f**) regenerated cotyledons, (**g**) regenerated shoots on rooting medium, (**h**) rooting initiation, and (**i**) acclimatization of the in vitro plants (Bars = 1 cm).

**Figure 2:**
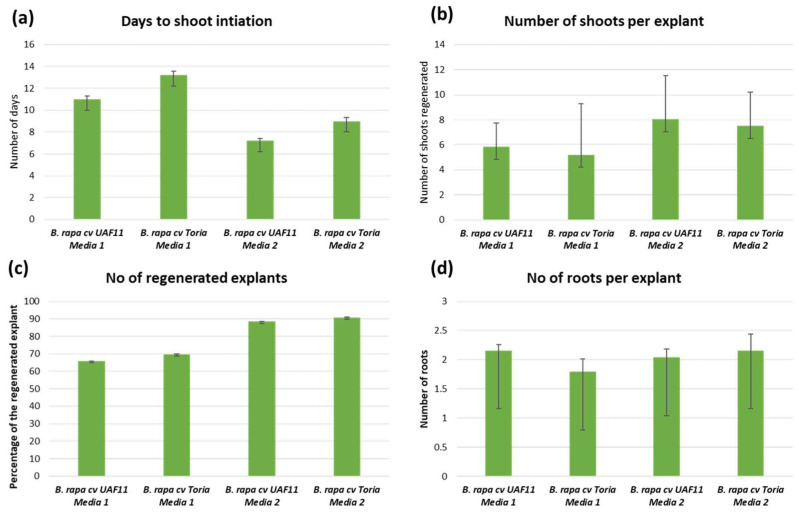
Mean comparison graphs for the *B. rapa* cv. UAF11, and *B. rapa* cv. Toria.

The germination percentage (Figure 1a) of UAF11 and Toria was 100% and 89%, respectively. Both cotyledons and hypocotyls formed calli, whereas (Figure 1) only cotyledons responded to shooting media, and hypocotyls started proliferating calli against both shooting media and were excluded from further experiments. Shoots were regenerated from the cut end of the petiole joined with cotyledon (Figure 1e). While separating the cotyledons from seedlings, meristem tissues were completely removed to avoid the growth of meristematic tissues. MS supplemented with 3 mg/L BAP + 0.4 mg/L NAA + 0.01 GA_3_ mg/L + 5 mg/L AgNO_3_ + 0.75 mg/L KI (SM2) produced plants from 90.8% explants of Toria and 88.4% of UAF11, whereas regenerants of MS supplemented with 2 mg/L BAP + 0.4 mg/L NAA + 0.01 mg/L GA_3_ + 5 mg/L AgNO_3_ + 0.75 mg/L KI (SM1) restricted to 69.6% in Toria and 65.8% in UAF11 (Figure 2c) (Table 1). Differences in regenerants of the same genotype for different media showed media to genotypic specific response for regeneration. However, shoot induction was faster on SM2 for UAF11, and the first shoot appeared after 7.2 days, whereas the same media took 9 days to induce the first shoot in the Toria genotype. The maximum time of shoot initiation was 13.2 days for Toria and 11 days for UAF11 (Figure 2a), (Table S1).

Similarly, the number of shoots per explant (NSE) also varied between media and genotypes (Figure 2b). SM1 showed no differences in shoots per explant for both genotypes and produced 5.8 mean shoots in UAF11, and 5.2 mean shoots in Toria. While SM2 produced the highest 8.04 mean NSE in UAF11 followed by 7.52 NSE in Toria (Figure 2b). However, differences were also not significant in SM2, which may be due to the less variation in hormonal concentration or genotypic/explant response towards regeneration (Figure 2). Although both SMs and genotypes did not show a range of results, this experiment was focused on optimizing the regeneration protocols in genotypes higher in agronomic values and inferior in fatty acid composition so that their genetic architecture could be altered via genome editing to edit fatty acid profile. When shoots attained optimum length, these were transferred to rooting media (RM) for root induction. Both RMs induced roots regardless of genotypes (Figure 2d).

### 2.2. In-Planta Transformation

**Figure 3:**
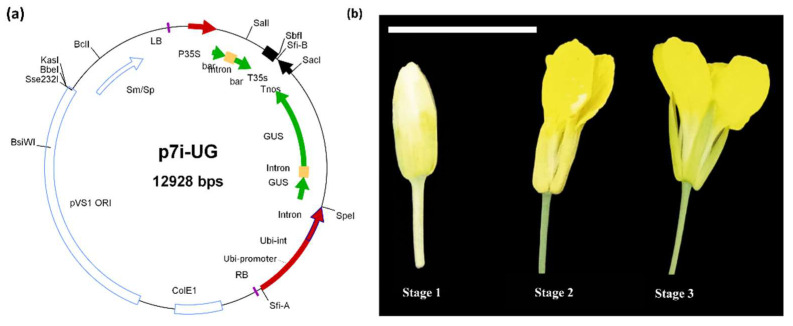
Expression vector and different flower stages used in *Agrobacterium*-mediated transformation. (**a**) P7i-UG construct harboring *GUS* reporter gene and *Bar* gene as a selectable marker, (**b**) flower stages attempted in *Agrobacterium*-mediated floral-dip transformation (Bar = 1 inch).

**Figure 4:**
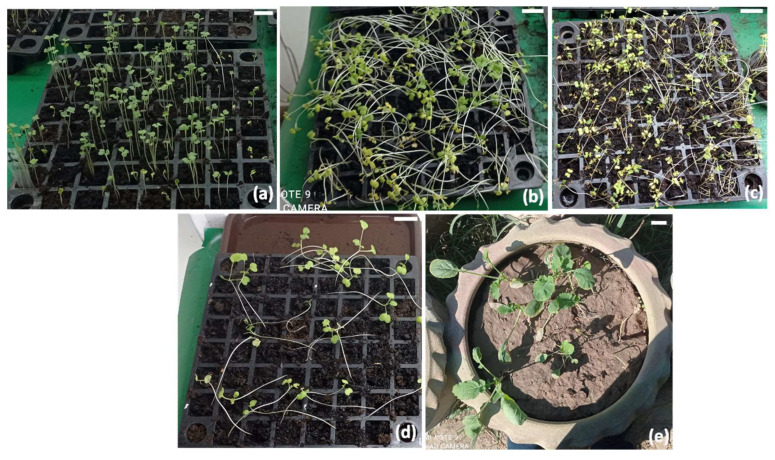
Transgenics selection with basta herbicide (Glufasinate-ammmonium), (**a**) germinated seeds after 14 days, (**b**) survived plants after 10 days of the first spray, (**c**) survived plants after10 days of the second spray, (**d**) plants survived the herbicide selection, and (**e**) selected transgenes in earthen pots (Bars = 1 inch).

**Figure 5:**
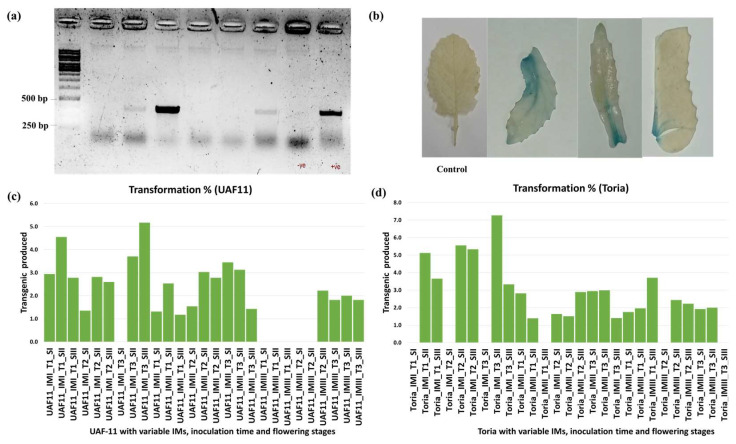
Screening for transgenics. (**a**) PCR conformation of visually screened plants (Transformed ones, -ve control, +ve control), (**b**) GUS histochemical assay of the identified positives (Bar = 1 inch), and (**c**,**d**) the Bar graph comparison of the different variables (IM: Induction medium, T: Cocultivation time, and S: Flower stage).

Floral buds, semi-opened flowers, and fully opened flowers were selected (Figure 3b), tagged accordingly, and treated with infection culture of transformed *Agrobacterium* (AGL-1) carrying P7i-UG (Figure 3a). Seeds of each treatment were harvested separately and grown under controlled conditions. After two weeks of germination (Figure 4a), the seedlings were sprayed with Glufosinate-ammonium (selection pressure, as P7i-UG carries bar as a selectable marker gene) to morphologically confirm transgenics (Figure 4b,c). Plants were sprayed thrice to reduce the chances of selecting the false positives (Figure 4d) and let the surviving plant grow in pots (Figure 4e). The surviving plants were further screened at the molecular level with GUS-specific primers, and plants showing desired PCR amplification were declared transgenic plants (Figure 5a). Among all combinations, a maximum of 7.8% of plants were shortlisted after PCR screening and declared as transgenics. Moreover, the leaves of transformed plants were subjected to GUS-histochemical analysis to observe the expression of GUS gene (Figure 5b).

Overall, floral buds gave minimum positive transformants in each induction media and time duration. For Toria genotype, floral buds gave minimum transformants on each IMs and time duration (Figure 5d). IMI and IMII (Table 2) produced a maximum of 7.1%, and 3% transformants, respectively, by inoculating the semi-opened flowers for 15 min (Figure 5d). Unlike IMI and IMII, IMIII (Table 2) produced a maximum of 3.8% transformants with 5 min of inoculation with fully opened flowers. Although IMIII showed less efficiency, its usefulness cannot be denied due to its ability of producing transformants by treating fully opened flowers, which is not observed in other IMs (Figure 5c,d). Interestingly, treating floral buds of UAF11 resulted in positive transformants, and floral buds treated with IMII for 15 min gave maximum 3.2% transformants (Figure 5c). However, in comparison to Toria, transformation efficiency was comparatively lower. In UAF11, 5.2% and 3.4% transformants were obtained with IMI and IMII by treating the fully opened flowers and flower buds for 15 min, respectively (Figure 5c) (Table S1). Both UAF11 and Toria genotypes have importance for genetic transformation, and methods cannot be used as an alternative to each other due to flower-specific response towards genetic transformation.

## 3. Discussion

### 3.1. Regeneration Response of B. rapa cv. UAF11 and Toria

Crop plants could be improved through conventional plant breeding or modern biotechnological tools in which genome editing is popular. Successful genome editing requires genotypic-specific regeneration protocols. The germination rate of UAF11 and Toria was 100% and 89%, respectively, without contamination, indicating an efficient sterilization protocol. Mainly this protocol was used to decontaminate the explants, but here, this protocol showed its efficiency in explant sterilization well [24,25]. Fourteen days old seedlings were used to isolate cotyledons and hypocotyls tissues after attaining 5 to 6 cm height as used in earlier studies [9,26,27,28,29,30] (Figure 1a). Cotyledons and hypocotyls have extensively been used for callus induction. BAP and NAA were used to induce callus, and both explants responded to these hormones (Figure 1c) [26]. However, concentrations and combinations of hormones could vary from species to species and cultivar to cultivar, which implies genotypic and specie-specific regeneration responses. The leaf explant of *B. rapa* could produce a higher percentage of callus [31] and regenerants. Variable BAP concentrations, along with 0.5 mg/L NAA, 0.01 mg/L GA_3,_ and 5 mg/L AgNO_3_ (Table 1), were used to induce shoots [32]. The cotyledons responded to both shooting media, but the average number of shoots increased to 8 shoots against enhanced BAP concentration (3 mg/L), whereas hypocotyls produced pseudo-callus against both shooting media [33]. However, shooting from hypocotyls of other *Brassica* species, *B. oleracea*, and *B. carinata* suggested genus and medium sensitivity towards direct regeneration [34,35,36]. The remaining experiment was carried out using cotyledonary leaves (Figure 1e–h).

Cotyledonary leaves have already been extensively used for regeneration in many *Brassica* species due to their responsiveness toward regeneration [26,29,37,38]. The genotypic genetic background greatly influences the regeneration in *Brassica* species [16,17]. UAF11 and Toria did not produce significant differences in number of shoots, and both genotypes produced maximum shoots from cotyledons on 3 mg/L BAP (Figure 1f) [26] but contrary to the finding of Goswami et al. [39] and Naz et al. [40] who reported optimum medium having 2 mg/L BAP for shoot regeneration. Several reports have shown genotype-specific regeneration, which is not in line with our findings, as both genotypes gave good response on the same medium [41,42]. The IBA is the widely used hormone for in-vitro rooting [43]. Enhancing IBA concentrations to 1 mg/L from 0.5 mg/L did not affect the number of roots and only one root (Figure 1i) [26,39].

### 3.2. In Planta Response of B. rapa cv. UAF11 and Toria

The *Agrobacterium*-mediated transformation was first attempted in *B. rapa* by Radke et al. [33]. Previously, the inefficiency of *B. rapa* to the regeneration was also reported [10,23], and complexities of the tissue culture protocols also made this technique difficult to follow. The regeneration of both cultivars was relatively low and only a small number of shoots were regenerated from the cotyledons. Therefore, regeneration requires significant modifications to use in genetic transformation experiments. Further, tissue culture needs technical and structural requirements to target a larger population size. However, in floral dip, Agrobacterium-mediated transformation maximum (7.3%) transformation percentage was observed, which was far better than the recently reported 0.1% success rate [21]. However, this efficiency is lower than the previously reported 16.25% and 10.83% transformants acquired through tissue culture-based genetic transformation [44,45].

The co-cultivation time and the induction medium composition influence the transformation efficiency [46]. The IMIII (MS + 500 µM Acetosyringone + 5 g Sucrose + 0.075% tween 20) with 15 min of flower dipping time in both genotypes produced maximum transformants. The addition of sucrose in IM helps to prolong the life of *Agrobacterium*, while Acetosyringone increases the virulence, and tween 20 improves the *Agrobacterium* attachment to the target cells. The addition of silwet in IMIII could be the reason for higher transformation efficiency, which is not present in the other two media (Table 2). Addition of silwet L-77 in media enhanced transformation efficiency [47,48]. The presence of acetosyringone also affects transformation efficiency, but the use of silwet L-77 along with acetosyringone could enhance the number of transformants.

The ovule is the main target in the *Agrobacterium*-mediated floral dip transformation method [49], so access to the ovule would ultimately affect the transformation efficiency. Fully opened flowers were the most efficient in UAF11 (5.2%), but in Toria (7.2%), half-opened flowers produced maximum transformants (Figure 2d). Therefore, differences in flower morphology could be the reason for different transformation efficiencies (Figure 3b).

## 4. Conclusions

The study aimed to optimize regeneration and transformation protocols for *B. rapa* genotypes. Hypocotyls were not responsive to regeneration and cotyledonary leaves produced regenerants but could not observe a range of results of shooting and rooting as well. However, this study provides the basis to improve regeneration efficiency in bilocular and multilocular genotypes, while *Agrobacterium*-mediated transformation via floral dip gave fruitful results. Maximum transformants of Toria genotype were acquired by treating the semi and fully opened flowers for 15 and 10 min, respectively, while UAF11 proved its usefulness by producing transformants through the floral bud treatment, which is not observed in Toria genotype. Current findings would help to understand or to improve both regeneration and transformation protocols by altering or modifying the current media compositions.

## 5. Materials and Methods

### 5.1. Optimization of the Regeneration Protocol

#### 5.1.1. In-Vitro Seeds Germination

The seeds of UAF11 and Toria were sourced from the Department of Plant Breeding and Genetics, University of Agriculture, Faisalabad, and both experiments were performed in Transformation Lab, CAS-AFS-UAF. Mature seeds were surface-sterilized with 70% ethanol (*v*/*v*) for 1 min, followed by 1-min washing with 0.5% mercuric chloride (*v*/*v*) [31]. Later, the seeds were washed with sterile distilled water and air-dried prior to in-vitro sowing (Table 1). Germination was attained in the dark at 25 ± 2 °C for 14 days. Seeds germination was counted by dividing the germinated seeds by the total number of sown seeds.

#### 5.1.2. Callus Induction, Shooting, and Shoot Proliferation

Fourteen days old in-vitro seedlings were excised to separate hypocotyls and cotyledons and transferred to callus induction medium as described by Bhalla and Singh [26]. After 10 days, explants were shifted to media with varying concentrations of BAP (Medium 1 (2 mg/L), and Medium 2 (3 mg/L)) to evaluate the shooting response. Subsequently, the regenerated explants were shifted to shoot proliferation media to increase the vigor of the originated shoots for the next 14 days.

#### 5.1.3. Rooting and Acclimatization

The regenerated shoots were excised from the base and placed on the rooting media (Table 2) with varying IBA concentrations (0.5 mg/L and 1 mg/L IBA (Indole-3-butyric acid)) for 4 weeks. Rooted plants were then acclimatized under ex-vivo conditions by transplanting the plant in cups filled with compost covered with a plastic bag for about 5 weeks in the growth room to avoid sudden environmental shock.

#### 5.1.4. Data Collection and Statistical Analysis

The experiment was performed under the Factorial CRD (Completely Randomized Design) with five experimental replications. The days to shooting, the number of shoots, and rooting data were collected by visually counting the number of the shoots and roots. The data were analyzed using Gen var package of R software [50].

### 5.2. Optimization of the Floral Dip Transformation Protocol

#### 5.2.1. Preparation of *Agrobacterium* Infection Culture

The *Agrobacterium* strain *AGL-1* harboring P7i-UG (Figure 3) expression vector was used to prepare the infection culture. The p7i-UG plasmid was purchased from DNA Cloning Service, Germany. The construct had *sp* gene for resistance against Spectinomycin [51]. The plasmid was transformed in *AGL-1* by preparing chemo-competent cells using the methodology given by DNA Cloning Services (https://dnacloning.com/agrobacteriumtransformation/, (accessed on 20 December 2022)). The transformed cells were screened for the desired plasmid on Rifampicin and Spectinomycin YEB plates and duly confirmed with PCR and double digestion.

#### 5.2.2. Preparation of Infection Culture

The confirmed isolated colony (*AGL-1*_p7i-UG) was isolated and inoculated in 10 mL YEB and cultured for 48 h at 28 °C in dark at 150 RPMs. The 5 mL (1:10) primary culture was used as a source for secondary culture for 4–6 h of incubation at 28 °C in the dark at 150 rpm. Bacteria were harvested in a refrigerated microcentrifuge at 5000 RPM for 5 min until OD600 reached 0.6, and the harvested *Agrobacterium* was resuspended in Induction Media (IM) (Table 1).

#### 5.2.3. In-Planta Transformation via Floral Dip

The *Agrobacterium* and infection media (IMI, IMII, and IMIII) [18,52] were prepared (Table 2). After 45 days of field sowing, *B. rapa* started flowering. The transformation was performed after 60 days when the flowering was at its peak. The experiment was performed at three stages of the flowering, green and closed buds (Stage 1), half-opened buds with exposed corolla (Stage 2), and fully opened flowers (Stage 3) (Figure 3). During the in-planta transformation attempt, only the target flowers were retained, while others were removed. The flowers were dipped in the infection medium (*Agrobacterium* strain *AGL-1* in 3 separate IM) for the different time durations of 5 min (T1), 10 min (T2), and 15 min (T3). Subsequently, the flowers were tagged (date, time, genotype, flower stage, and infection medium type) from the base. The flowers were then covered with butter paper bags to limit cross-pollination.

#### 5.2.4. Screening of Transgenics through Selectable Marker

The expected transformed seeds were sown in trays containing compost under growth room conditions, and after 14 days of germination, plants were sprayed with basta herbicide, (Glufosinate ammonium @150 mg/L), as P7i-UG contains *bar* gene, and this process was again repeated after 10 days. Surviving plants were selected for further molecular analysis.

#### 5.2.5. Confirmation of the Transgenic Plants with PCR

The leaves of putative transgenic plants were removed, DNA was extracted following the protocol by Kidwell and Osborn [26], and isolated DNA was checked for quality and quantity standards. The *GUS* gene-specific primers were used for PCR amplification of the inserted cassette, F-P7i-*GUS* AATAACGGTTCAGGCACAGCACAT and R-P7i-*GUS* GCTCGACTGGGCAGATGAACA. The PCR was performed as 35 cycles, 95 °C for 1 min, 58 °C for 30 s, 72 °C for 1 min and final extension at 72 °C for 10 min.

Finally, the plants showing desired PCR amplification (396 bp) were further tested for observing the GUS expression in leave tissues via GUS-histochemical analysis.

The leaves of the selected plants were submerged in 10 mL X-GLUC regents (0.5 M MES, 200 mM NaPO_4_, and X-GLUC Stain) overnight at 37 °C followed by submerging in 70% ethanol for 6 h to remove chlorophyll content.

## Figures and Tables

**Table 1:** Growth regulators used in in-vitro regeneration experiment.

Components	Sowing Medium	Callus Induction Medium	Shooting Medium	Shoot Proliferation Medium	Rooting Medium
**MS**	4.43 g/L	4.43 g/L	4.43 g/L	4.43 g/L	4.43 g/L
**Sucrose**	20 g/L	20 g/L	20 g/L	20 g/L	20 g/L
**Phytagel**	4 g/L	4 g/L	4 g/L	4 g/L	4 g/L
**BAP**	-	0.75 mg/L	Variable	Variable	-
**NAA (1-Naphthalene Acetic Acid)**	-	0.4 mg/L	0.4 mg/L	-	-
GA * (Gibberellic acid)	-	0.01 mg/L	0.01 mg/L	-	-
**IBA**	-	-	-	-	Variable
AgNO_3_ *	5 mg/L	5 mg/L	5 mg/L	5 mg/L	-
**Adenine hemisulfate**	-	-	-	40 mg/L	-
**PVP (Polyvinylpyrrolidone)**	-	-	-	500 mg/L	-
**KI (Potassium Iodide)**	-	0.75 mg/L	0.75 mg/L	0.75 mg/L	0.37 mg/L

The pH was maintained at ~5.8 ± 1 with NaOH and HCL (* labels the heat-sensitive chemicals in this table).

**Table 2:** Composition of the IM used in the experiment.

Components	IM-I	IM-II	IM-III
**MS**	Half MS	MS	MS
**Acetosyringone**	-	-	100 μM
**Tween 20**	-	-	0.075%
**Silwet L-77**	500 μL	-	-
**Sucrose**	5%	3%	5%
**BAP**	-	0.5 μM	-

BAP = 6-benzylaminopurine, and MS (PhytoTech M254).

## Data Availability

Not applicable.

## Supplementary Materials

The supplementary data supporting the study is accessible at https://www.mdpi.com/article/10.3390/plants12010161/s1, Table S1: Mean data of the tissue culture (Figure 2). Table S2: Standard errors (SE) of the Table S1. Table S3: Percentage of the transformants observed under different treatments.
